# Gynæcology

**Published:** 1879-03

**Authors:** 


### GYNÆCOLOGY.

### PECULIAR ACTION OF TINCTURE OF IODINE ON THE CERVIX UTERI. (*Bulletin de Thérap.*, August 30th, 1878.)

M. Laboulbène says, that tincture of iodine colors the healthy cervix a uniform, dark brown. But if slight ulcerations or granulations or vegetations exist, the affected patch is colored yellow. This may afford a delicate means of diagnosis. If the cervix be large and the tincture produce patches of yellow color, a neoplasm may be apprehended, with approaching ulceration in the points least colored. Under any circumstances as a healthy state is approached, the discoloration caused by the iodine assumes more and more the dark brown hue.

